# Eliminating the "Pitfalls" of chronic indwelling central venous access device placement in cancer patients by utilizing a venous cutdown approach and by selectively and appropriately utilizing intraoperative venography

**DOI:** 10.1186/1477-7800-4-16

**Published:** 2007-07-09

**Authors:** Stephen P Povoski

**Affiliations:** 1 Section of Surgical Oncology, Department of Surgery, Arthur G. James Cancer Hospital and Richard J. Solove Research Institute and Comprehensive Cancer Center, The Ohio State University, Columbus, Ohio, 43210, USA

## Abstract

There are several very obvious and simple solutions for eliminating the "Pitfalls" and for minimizing the risk of occurrence of any perioperative complications associated with placement of chronic indwelling central venous access devices in cancer patients. The first is the utilization of a venous cutdown approach, such as the cephalic vein or the external jugular vein, which essentially eliminates potentially life-threatening perioperative complications, such as pneumothorax and injury to the great vessels (with or without associated hemothorax). The second is the selective and appropriate utilization of intraoperative venography for defining the central venous anatomy and for providing a venous roadmap in those particularly challenging cases in which difficulties are encountered during chronic indwelling central venous access device placement.

## Commentary

The placement of chronic indwelling central venous access devices in cancer patients remains an essential service not infrequently provided by many practicing surgical oncologists [1]. Although generally straight-forward and seemingly simple, such procedures can be highly challenging to even the most experienced surgeon secondary to variations in body habitus, pre-existing co-morbid medical conditions, altered venous anatomy, and inadequate volume status of the cancer patient. Therefore, having a basic fund of knowledge about alternative and supplemental strategies for chronic indwelling central venous access device placement can be essential to maximizing the success of device placement and to minimizing the risk of occurrence of any potential perioperative complications.

In a recently published article featured in International Seminars in Surgical Oncology on June 5, 2007, entitled "Pitfalls in Portacath location using the landmark technique: case report", Wyles et al [2] set out to describe "a case report that highlights potential difficulties in identifying the catheter position and consider the limitations of common methods of confirming the catheter location". It appears that the authors did recognize the immediate difficulties that they were faced with in this particular challenging case in which placement of the chronic indwelling central venous access device was accomplished by way of a percutaneous subclavian vein approach. Likewise, in their report, they discussed several manners for how these encountered difficulties could have been addressed. However, they failed to discuss two very obvious and simple alternative solutions that would have eliminated the "Pitfalls" and would have minimized the risk of occurrence of any potential perioperative complications. These simple alternative and supplemental solutions are: (1) consideration of a venous cutdown approach and (2) selective and appropriate utilization of intraoperative venography.

Two particularly useful venous cutdown approaches for placement of chronic indwelling central venous access devices in cancer patients are the cephalic vein cutdown approach [3,4] and the external jugular vein cutdown approach [4]. The cephalic vein cutdown approach was first described in 1976 by Heimbach and Ivey [5]. The external jugular vein cutdown approach was first described in 1966 by Rams et al [6]. Both these superficially located veins represent convenient venous conduits that can be easily and safely accessed by way of a superficial venous cutdown approach for providing an easily accessible peripheral point of entry into the central venous system. The combined success of sequentially utilizing the cephalic vein cutdown approach and the external jugular vein cutdown approach in appropriately selected cancer patients has previously been shown to be greater than 99% [4]. The use of these venous cutdown approaches completely eliminates the chance of inadvertent cannulation of the arterial system, as was obviously an ongoing concern expressed by Wyles et al in their recent case report [2]. Likewise, these cutdown approaches essentially eliminate the risk of potentially life-threatening perioperative complications, such as pneumothorax and injury to the great vessels (with or without associated hemothorax) [3,4]. Such potentially life-threatening perioperative complications are well-known to be associated with the percutaneous venipuncture approach to the subclavian vein or internal jugular vein using the modified Seldinger technique [1]. Whereas, long-term complications, such as catheter-related bacteremia, device site infections, and deep venous thrombosis, appear to be identical for both the venous cutdown approach and the percutaneous venipuncture approach [1,3,4].

The venous cutdown approach represents an easy, safe, and highly useful avenue for selectively and appropriately performing intraoperative venography during challenging central venous access cases [7]. Such a venous cutdown approach allows for performing intraoperative venography with the injection of contrast at the point of entry into the most peripheral venous conduit (i.e., cephalic vein or external jugular vein). Whereas, the percutaneous venipuncture approach allows for the injection of contrast at the point of entry into the most peripheral venous conduit (i.e., subclavian vein or internal jugular vein) only at an early phase in the modified Seldinger technique when the venopunture needle is still in place or even as far into the procedure as when the dilator and peel-away sheath apparatus are still in place (with or without the catheter in place). However, once the catheter has been passed through the peel-away sheath and advanced to its anticipated central venous location at the junction of the superior vena cava and right atrium, and the peel-away sheath has been subsequently peeled back off from the catheter, then intraoperative venography, in a practical sense, can only be performed through the tip of the already centrally placed catheter. At any time prior to peeling back the peel-away sheath, the catheter can be positioned as peripherally as possible for successful intraoperative venography since such a catheter tip position represent the most peripheral point of entry into the cannulated venous conduit (i.e., subclavian vein or internal jugular vein). While Wyles et al [2] stated that they attempted injection of venous contrast "down the catheter" and that it was "unhelpful in distinguishing arterial from venous catheterization" because the "contrast dissipated so rapidly", they simply failed to recognize that their optimal contrast visualization would have been best obtained by the injection of contrast at the most peripheral point of entry into the subclavian vein, rather than through the tip of the catheter once the catheter was positioned in its most central location at the junction of the superior vena cava and right atrium (independent of whether the peel-away sheath was still present or not). Obviously, as a result of the central location of the tip of the catheter, rapid contrast dissipation (i.e., washout) and the inability to obtain a readable intraoperative fluoroscopic image occurred. This would not be unexpected in such a centrally-placed location within the central venous system. The resultant rapid contrast dissipation (i.e., washout) and the inability to obtain a readable intraoperative fluoroscopic image would have occurred with the tip of the catheter in such a central location independent of whether the catheter was truly within the venous system or whether the catheter was inadvertently within the arterial system. Theoretically, only augmentation of the injection volume and injection rate of the contrast by some sort of mechanical power injection device could have aided at that point for distinguishing a central venous location from a central arterial location. Therefore, their utilization of intraoperative venography was terribly flawed and resultantly (but not unexpectedly) inconclusive.

Wyles et al [2] inferred that perioperative venous ultrasound can be a potentially useful adjunct to the percutaneous venipuncture approach. While this fact is absolutely true, it has also been previously shown that up to 50% of cancer patients with venous abnormalities identifiable on intraoperative venography had no recognizable venous abnormalities on perioperative venous ultrasound [7]. This is most simply explained by the fact that many venous abnormalities are located in a more central location within the venous system (i.e., along the medial segment of the subclavian vein, along the innominate vein, or within the superior vena cava), thus representing segments of the central venous anatomy which are not ideally accessible to visualization by perioperative venous ultrasound [7]. Thus, the selective use of intraoperative venography, by either a venous cutdown approach or a percutaneous venipuncture approach, which is appropriately performed at the most peripheral point of entry into the central venous system, can be an invaluable tool to the surgeon for defining the central venous anatomy and for providing a venous roadmap in those particularly challenging cases in which difficulties are encountered during chronic indwelling central venous access device placement [7].

In conclusion, consideration of a venous cutdown approach, as well as selective and appropriate utilization of intraoperative venography, represent very obvious and simple alternative solutions for eliminating the "Pitfalls" and for minimizing the risk of occurrence of any perioperative complications associated with placement of chronic indwelling central venous access devices in cancer patients.

## Competing interests

The author(s) declare that they have no competing interests.

## Authors' contributions

The author organized, wrote, and edited all aspects of this manuscript. He approved the final version of this manuscript.
